# Long-term Real-world Survival Outcomes with Dual Immune Checkpoint Blockade in Synchronous Metastatic Renal Cell Carcinoma: Implications for the Design of Prospective Cytoreductive Nephrectomy Trials

**DOI:** 10.1016/j.euros.2025.12.004

**Published:** 2025-12-19

**Authors:** Leo Bickley, Elisabeth E. Fransen van de Putte, Luna van den Brink, Laura Marandino, Johannes C. van der Mijn, Sofie Wilgenhof, Johannes V. van Thienen, John B.A.G. Haanen, Ekaterini Boleti, Thomas Powles, Patricia J. Zondervan, Niels M. Graafland, Zayd Tippu, Samra Turajlic, Axel Bex

**Affiliations:** a Specialist Centre for Kidney Cancer, Department of Urology, Royal Free London NHS Foundation Trust, London, UK; b Cancer Dynamics Laboratory, Francis Crick Institute, London, UK; c Department of Urology, Netherlands Cancer Institute, Amsterdam, The Netherlands; d Department of Urology, Amsterdam UMC, University of Amsterdam, Amsterdam, The Netherlands; e Department of Imaging & Biomarkers, Cancer Centre Amsterdam, Amsterdam, The Netherlands; f Renal and Skin Units, Royal Marsden NHS Foundation Trust, London, UK; g Department of Medical Oncology, Netherlands Cancer Institute, Amsterdam, The Netherlands; h Specialist Centre for Kidney Cancer, Department of Medical Oncology, Royal Free London NHS Foundation Trust, London, UK; i Barts Cancer Institute, Queen Mary University, London, UK; j Renal Cancer Network, Amsterdam, The Netherlands; k Division of Surgery and Interventional Science, University College London, London, UK

**Keywords:** Renal cell carcinoma, Clear-cell metastatic renal cell carcinoma, Immune checkpoint inhibitors, Ipilimumab, Nivolumab, Nephrectomy, Retrospective studies

## Abstract

**Background and objective:**

Patients with synchronous metastatic renal cell carcinoma (s-mRCC) increasingly undergo systemic therapy with their primary tumour in situ. We report long-term survival outcomes and deferred cytoreductive nephrectromy (dCN) rates in an unselected real-world s-mRCC cohort of patients treated with nivolumab + ipilimumab.

**Methods:**

This was a retrospective cohort study of 287 patients with s-mRCC treated with nivolumab + ipilimumab between 2018 and 2024 at five European institutions. Data were collected for International mRCC Database Consortium (IMDC) risk, overall survival (OS), progression-free survival (PFS), treatment responses, and dCN rates.

**Key findings and limitations:**

At median follow-up of 23.5 mo, median OS was 29.0 mo (95% confidence interval [CI] 20.1–36.2) for the overall cohort (*n* = 287), and 49.8 mo (95% CI 33.1–not reached) for the intermediate-risk group (*n* = 144, 50%) versus 16.3 mo (95% CI 13.5–26.3) for the poor-risk group (*n* = 143, 50%; hazard ratio [HR] 0.50, 95% CI 0.35–0.71; *p* < 0.001). IMDC risk was the only significant baseline multivariable predictor for both OS and PFS. Among patients with a complete or near-complete response (CR/nCR) at metastatic sites, there was no significant difference in OS between subgroups with dCN owing to the depth of response (*n* = 27) and without dCN (*n* = 23; HR 1.00, 95% CI 0.29–3.47; *p* > 0.9).

**Conclusions and clinical implications:**

Real-world treatment of s-mRCC with nivolumab + ipilimumab yields encouraging OS, especially in patients with intermediate IMDC risk and CR/nCR at metastatic sites. Trials investigating dCN following immunotherapy may be impacted by this lower-than-expected event rate, which could potentially affect their estimated sample sizes.

**Patient summary:**

We looked at outcomes for patients with metastatic kidney cancer who were treated with immunotherapy while their kidney tumour was still in place. Patients who responded well to immunotherapy were likely to survive for a long time, whether or not they then had surgery to remove their kidney tumour. Our results will help in the design of analyses for clinical trials that are already testing the role of delayed surgery for metastatic kidney tumours.

## Take Home Message

Real-world treatment of synchronous metastatic renal cell carcinoma with nivolumab + ipilimumab yields encouraging overall survival. Exceptional responders show excellent survival, even without deferred cytoreductive nephrectomy. Currently recruiting trials of deferred cytoreductive nephrectomy are likely to underestimate eventual survival in their control arms.

## 1 Introduction

Management of synchronous metastatic renal cell carcinoma (s-mRCC), defined as the presence of a primary tumour in situ when metastatic disease is diagnosed, has evolved significantly in recent decades, with notable changes in incidence, systemic treatment strategies, and the role of cytoreductive nephrectomy (CN) [1].

Until the early 2000s, upfront CN before systemic therapy was considered a key intervention in s-mRCC for alleviating symptoms, reducing the tumour burden, and potentially augmenting the efficacy of subsequent systemic therapy. This approach was supported by the SWOG-8949 [2] and EORTC-30947 [3] trials, which demonstrated a survival benefit when upfront CN was added to interferon treatment.

However, in the subsequent VEGFR-targeted treatment era, the role of upfront CN became less clear. The CARMENA [4] and SURTIME [5] trials demonstrated that upfront CN did not provide a survival benefit for patients for whom sunitinib therapy was indicated, and that some patients could instead benefit from deferred CN (dCN).

The modern era of first-line mRCC treatment is centred on immune checkpoint inhibitor (ICI)-based combination regimens [1]. In this context, current guidelines [1], [6] recommend that patients with s-mRCC requiring systemic therapy should receive it upfront, with dCN then considered if a durable response is achieved. Patient selection for dCN is critical, and should involve a multidisciplinary approach that takes performance status, metastatic burden, and primary tumour symptoms into account.

However, a considerable limitation to guidelines regarding dCN in the ICI era is the lack of prospective randomised trial data. The role of dCN is currently being evaluated in the NORDIC-SUN and PROBE trials [7], [8], but trial design in this setting has well-recognised challenges [9]. Real-world data on long-term outcomes would help to guide statistical considerations. However, although numerous articles have provided survival data for patients with s-mRCC who undergo dCN [10], [11], [12], [13], [14], [15], [16], [17], [18], [19], these are for inherently highly selected cohorts, as patients who achieved durable responses but did not undergo dCN were excluded.

In this retrospective study, we expand on and report long-term survival data for a previously published [20], [21] real-world cohort of patients with s-mRCC treated with the commonly used ICI-based regimen nivolumab + ipilimumab (N + I) at five European centres. The unselected population includes patients with and without subsequent dCN. We also use our findings to project survival for patients meeting the inclusion criteria for PROBE and NORDIC-SUN.

## 2 Patients and methods

We gathered consecutive retrospective data for all patients with s-mRCC treated with N + I at five European centres between 2018 and 2024 (Table 1) in accordance with local ethics procedures. Patients treated with N + I for a diagnosis of mRCC were identified from local electronic records, and data on patient characteristics, clinicopathological status, and treatment interruptions were collected. Patients who had either metachronous disease (including prior nephrectomy), prior systemic therapy, or upfront nephrectomy for this episode of RCC were excluded. The primary objective was to determine overall survival (OS). Secondary analyses included progression-free survival (PFS), best response, serial size measurements for the primary tumour, and details of dCN if applicable.

**Table 1:** Baseline characteristics of the overall cohort (*n* = 287) and rate of deferred cytoreductive nephrectomy

Parameter	Result
Institution, *n* (%)	
Netherlands Cancer Institute-Antoni van Leeuwenhoek Hospital (NL)	105 (37)
Royal Marsden Hospital (UK)	57 (20)
Royal Free Hospital (UK)	50 (17)
Amsterdam University Medical Centre (NL)	43 (15)
St. Bartholomew’s Hospital (UK)	32 (11)
Median age, yr (interquartile range)	63 (56–71)
Male sex, *n* (%)	202 (70)
Eastern Cooperative Oncology Group performance status, *n* (%)	
0	113 (39)
1	132 (46)
≥2	42 (15)
International Metastatic RCC Database Consortium (IMDC) risk category, *n* (%)	
Intermediate (1–2)	144 (50)
Poor (≥3)	143 (50)
Median primary tumour size, mm (interquartile range)	95 (75–124)
Number of metastatic sites, *n* (%)	
1 site	75 (26)
2 sites	100 (35)
3 sites	65 (23)
≥4 sites	47 (16)
Location of metastatic sites, *n* (%)	
Lung	201 (70)
Lymph node	158 (55)
Bone	118 (41)
Adrenal	58 (20)
Liver	40 (14)
Brain	27 (9.4)
Pancreas	8 (2.8)
Histological subtype, *n* (%)	
Clear cell	230 (80)
Clear cell with sarcomatoid and/or rhabdoid features	21 (7.3)
Sarcomatoid and/or rhabdoid features, with underlying subtype not identifiable	11 (3.8)
Unclassified	15 (5.2)
Papillary	7 (2.4)
Other a	3 (1.0)
Chromophobe	1 (0.35)
Deferred cytoreductive nephrectomy, *n* (%)	49 (17)
Main clinical reason for deferred cytoreductive nephrectomy, *n* (%)	
Complete response at metastatic sites	19 (39)
Near complete response at metastatic sites	8 (16)
Partial response at metastatic sites	3 (6.1)
Progressive disease in the primary tumour only	11 (22)
Symptomatic control of the primary tumour	8 (16)

RCC = renal cell carcinoma.

a Other histological subtypes were MiT (microphtalmia-associated transcriptional factor) translocation (*n* = 1), fumarate hydratase deficiency (*n* = 1), and clear-cell papillary RCC (*n* = 1).

Data for radiological responses at metastatic sites and at the primary tumour were collected and categorised according to Response Evaluation Criteria in Solid Tumours version 1.1 (RECIST v1.1) [22] as progressive disease (PD), stable disease (SD), partial response (PR), or complete response (CR), with an additional category of near-CR (nCR) defined as a >80% reduction at metastatic sites [13]. The primary tumour size was calculated manually as the long-axis measurement from each on-treatment imaging study, with imaging time points accepted within ±4 wk of the interval stated. CR or nCR at metastatic sites was considered an exceptional response (ER). The dCN indication was recorded and classified in terms of whether it was to facilitate no evidence of disease (NED) in ER patients, or to control primary tumour progression or symptoms such as haematuria and pain in any patients (ER, PR, or SD at metastatic sites).

OS and PFS, including landmark analyses, were estimated via the Kaplan-Meier method, with Cox regression to test associations; further details are provided in the Supplementary material. Association between primary tumour downsizing at 3 mo and subsequent achievement of ER (including patients at 3 mo without death, progression, or previous ER) was tested using logistic regression.

Data analysis was performed using RStudio 2025.05.0+496 (Posit, Boston, MA, USA). A *p* value of <0.05 was considered statistically significant. Results for continuous and categorical variables were compared between groups using a Mann-Whitney *U* test and Fisher’s exact test, respectively.

## 3 Results

Data were collected for 287 patients with s-mRCC treated with N + I. Baseline characteristics are listed in Table 1.

**Fig. 1:**
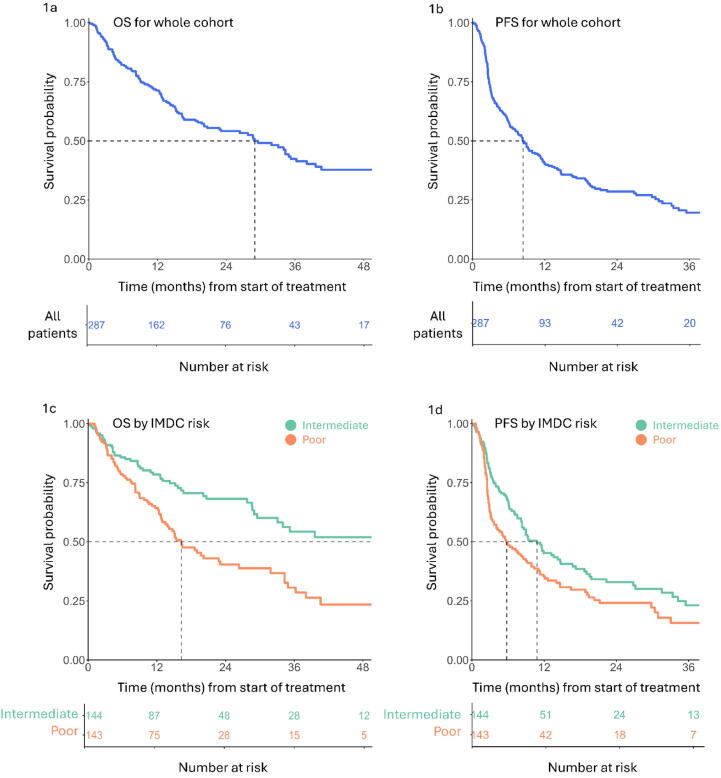
Kaplan-Meier plots of overall survival (OS) and progression-free survival (PFS) over time for the overall cohort and stratified by IMDC risk group. There were 129 OS events and 199 PFS events. Dotted lines at a survival probability of 0.50 represent median survival. Plots are truncated when the number at risk in any group was <5. IMDC = International Metastatic Renal Cell Carcinoma Database Consortium.

Kaplan-Meier survival analysis was performed for OS and PFS for the overall cohort (Fig. 1A,B and Table 2) and for groups stratified by clinical variables at baseline (Table 3); median follow-up was 23.5 mo for event-free patients. Median drug exposure, with documented treatment breaks and discontinuations taken into account, was 5.3 mo (interquartile range [IQR] 2.5–12 mo) for the overall cohort and 10.9 mo (IQR 5.4–17 mo) for patients with an overall response at metastatic sites.

**Table 2:** Cox regression results for baseline characteristics associated with OS a

Variable	Median OS,	Univariable regression		Multivariable regression b	
	mo (95% CI)	HR (95% CI)	*p* value	aHR (95% CI)	*p* value
Overall cohort (*n* = 287)	29.0 (20.1–36.2)	–	–	–	–
**IMDC risk group**					
Intermediate-risk (*n* = 144)	49.8 (33.1–NR)	0.50 (0.35–0.71)	<0.001	0.53 (0.36–0.77)	<0.001
Poor risk (*n* = 143)	16.3 (13.5–26.3)	Reference		Reference	
**Age**					
<75 yr (*n* = 252)	28.7 (20.1–36.2)	1.25 (0.71–2.23)	0.4	1.24 (0.69–2.20)	0.5
≥75 yr (*n* = 35)	39.6 (15.2–NR)	Reference		Reference	
**RCC histology**					
ccRCC with no S/R features (*n* = 230)	34.0 (27.8)–49.8)	Reference		Reference	
S/R features, AUS (*n* = 32)	16.3 (11.1–NR)	1.43 (0.85–2.40)	0.2	1.17 (0.69–1.98))	0.6
Other RCC histology (*n* = 25)	15.1 (12.6–NR)	1.65 (0.96–2.85)	0.07	1.67 (0.97–2.90)	0.07
**Number of metastatic sites**					
1 site at baseline (*n* = 75)	33.1 (23.1–NR)	Reference		Reference	
2 sites at baseline (*n* = 100)	34.3 (28.6–NR)	0.95 (0.60–1.51)	0.8	0.93 (0.59–1.49)	0.8
3 sites at baseline (*n* = 65)	26.3 (13.0–NR)	1.20 (0.72–1.98)	0.5	1.03 (0.62–1.72)	0.9
≥4 sites at baseline (*n* = 47)	14.3 (10.3–NR)	1.75 (1.04–2.94)	0.03	1.58 (0.94–2.67)	0.08

AUS = any underlying subtype; CI = confidence interval; HR = hazard ratio; aHR = adjusted HR; IMDC = International Metastatic RCC Database Consortium; NR = not reached; OS = overall survival RCC = renal cell carcinoma; ccRCC = clear cell RCC; S/R = sarcomatoid/rhabdoid.

a Variables for univariable and multivariable analyses were selected on clinical grounds and are all presented here, regardless of statistical significance.

b The overall likelihood ratio for multivariable analysis was 23.1 with seven degrees of freedom (*p* = 0.002). Median follow-up for OS was 23.5 mo.

**Table 3:** Cox regression analyses for baseline characteristics associated with PFS a

Variable	Median PFS,	Univariable regression		Multivariable regression b	
	mo (95% CI)	HR (95% CI)	*p* value	aHR (95% CI)	*p* value
Overall cohort (*n* = 287)	8.4 (6.6–11.3)	–	–	–	–
**IMDC risk group**					
Intermediate-risk (*n* = 144)	10.8 (8.3–16.7)	0.71 (0.54–0.94)	0.02	0.73 (0.54–0.97)	0.03
Poor risk (*n* = 143)	5.8 (3.7–9.9)	Reference		Reference	
**Age**					
<75 yr (*n* = 252)	7.7 (5.9–9.4)	1.41 (0.90–2.22)	0.1	1.37 (0.87–2.17)	0.2
≥75 yr (*n* = 35)	12.6 (9.9–NR)	Reference		Reference	
**RCC histology**					
ccRCC without S/R features (*n* = 230)	9.0 (7.5–11.6)	Reference		Reference	
S/R features, AUS (*n* = 32)	6.2 (4.6–NR)	1.08 (0.68–1.70)	0.7	0.98 (0.61–1.57)	0.9
Other histology (n = 25)	5.4 (2.8–NR)	1.13 (0.70–1.84)	0.6	1.13 (0.69–1.85)	0.6
**Number of metastatic sites**					
1 site at baseline (*n* = 75)	10.3 (8.2–26.8)	Reference		Reference	
2 sites at baseline (*n* = 100)	11.0 (7.5–18.7)	1.01 (0.70–1.46)	>0.9	1.02 (0.70–1.47)	0.9
3 sites at baseline (*n* = 65)	6.3 (3.4–14.8)	1.17 (0.78–1.76)	0.5	1.10 (0.73–1.66)	0.7
≥4 sites at baseline (*n* = 47)	4.3 (2.7–9.0)	1.85 (1.22–2.80)	0.004	1.76 (1.16–2.68)	0.008

AUS = any underlying subtype; CI = confidence interval; HR = hazard ratio; aHR = adjusted HR; IMDC = International Metastatic RCC Database Consortium; NR = not reached; PFS = progression-free survival RCC = renal cell carcinoma; ccRCC = clear cell RCC; S/R = sarcomatoid/rhabdoid.

a Variables for univariable and multivariable analyses were selected on clinical grounds and are all presented here, regardless of statistical significance.

b The overall likelihood ratio for multivariable analysis was 16.8 with seven degrees of freedom (*p* = 0.02).

Median OS for the overall cohort was 29.0 mo, with a 4-yr landmark OS rate of 38% (95% confidence interval [CI] 31–47%). The most significantly predictive covariate for OS was IMDC risk group, with median OS of 49.8 mo for the intermediate-risk group versus 16.3 mo for the poor-risk group (adjusted HR 0.53; *p* < 0.001; Table 2 and Fig. 1C). This corresponded to landmark 4-yr OS rates of 52% (95% CI 42–65%) and 23% (95% CI 15–37%), respectively. A significant association between the number of metastatic sites and worse PFS was only observed for patients with metastases at four or more different organ sites (Table 3).

Response to N + I was assessed according to RECIST v1.1. Primary and metastatic sites were considered separately; a dissociated response to ICI is common [23] and affects decision-making for dCN. The objective response rate (ORR) was 44% for metastatic sites and 30% for the primary tumour, with corresponding CR rates of 13% and 0%. Among responders, the median time to best response was 3.9 mo (IQR 2.8–6.1) for metastatic sites and 4.8 mo (IQR 2.7–6.4) for the primary tumour, with a median duration of best response of 8.0 mo (95% CI 5.3–9.9) and 6.4 mo (95% CI 2.9–12.0), respectively. The proportion of patients who had PD at metastatic sites at first follow-up disease assessment or who had died before this assessment was 33% (41% of the poor-risk group, 25% of the intermediate-risk group; *p* = 0.003, Mann-Whitney *U* test) at a median of 2.5 mo (IQR 1.8–2.9 mo) after treatment initiation.

**Fig. 2:**
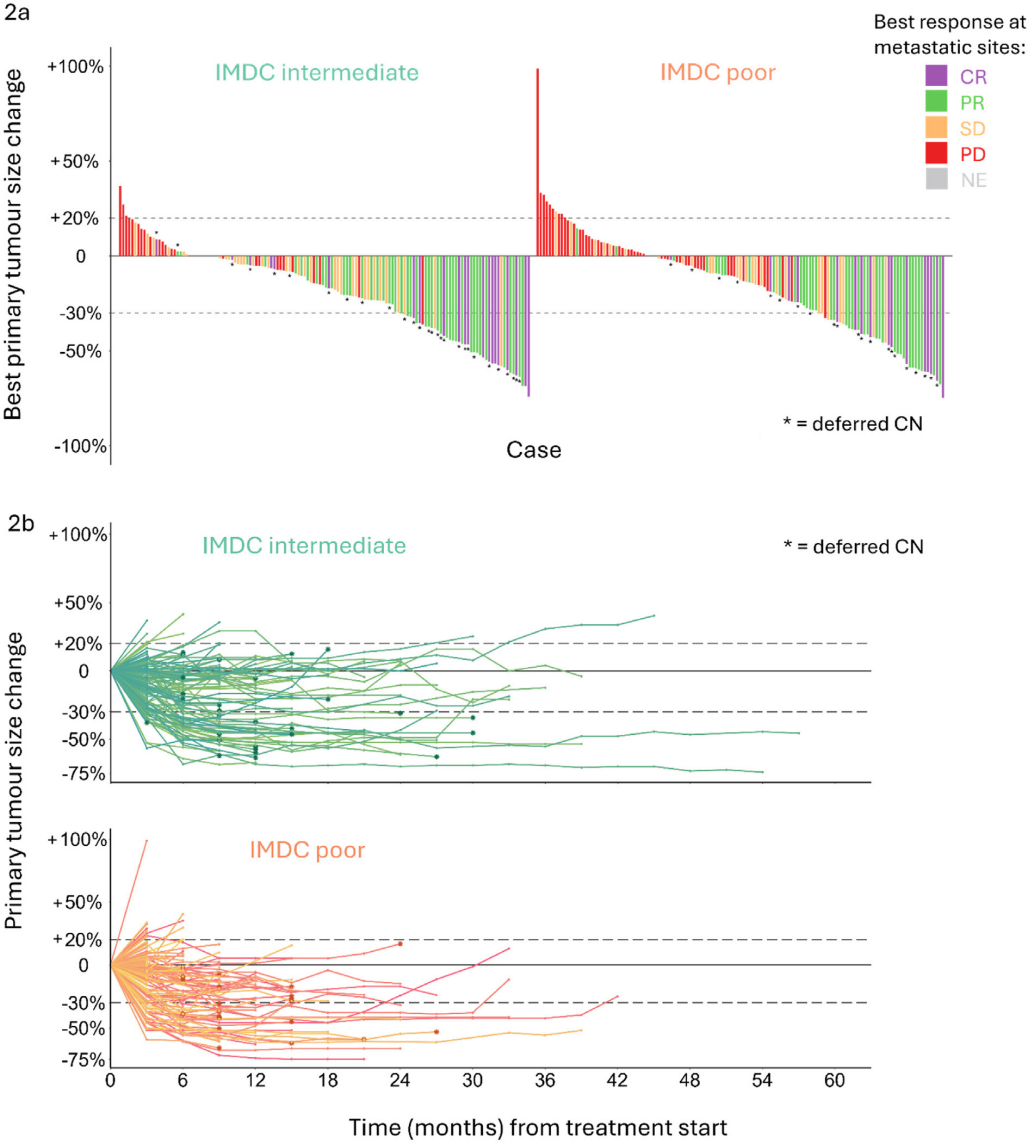
Response in the primary tumour, stratified by IMDC risk category. (A) Waterfall plot depicting the best response in the primary tumour for each case as the percentage change from baseline. Cases are coloured according to the best metastatic response according to Response Evaluation Criteria in Solid Tumours version 1.1. (B) Spider plots depicting the primary tumour response over time case (to progression, death, or last follow-up) as the percentage change from baseline; colours differ within the IMDC categories for the purpose of visual separation only. CR = complete response; PR = partial response; SD = stable disease; PD = progressive disease; NE = not evaluable; CN = cytoreductive nephrectomy; IMDC = International Metastatic Renal Cell Carcinoma Database Consortium.

The change in size for the primary renal tumour per case is depicted in Fig. 2. The median best change in size for the primary renal tumour was −16% in the intermediate-risk group versus −13% in the poor-risk group (*p* = 0.4, Mann-Whitney *U* test); the primary tumour PR rate was 30% in both groups. There was a significant association between ORR for the primary tumour and ORR for metastatic sites (odds ratio 19.9, 95% CI 9.58–45.1; *p* < 0.001, Fisher’s exact test).

According to landmark analysis, the extent of primary tumour downsizing at 3 mo (per 10% increment) was already associated with subsequent OS (HR 0.69, 95% CI 0.62–0.77; *p* < 0.001) and with subsequent ER attainment at metastatic sites (odds ratio 1.71, standard error 0.10; *p* < 0.001). The timing of the primary tumour response was not significantly associated with OS according to a 6-mo landmark analysis comparing late responders (primary tumour PR only by 6 mo, n = 32) to early responders (primary tumour PR by 3 mo, *n* = 43; HR for late vs early: 0.54, 95% CI 0.20–1.46; *p* = 0.2). There was no evidence that larger primary tumours (third tertile at baseline [24]) showed greater downsizing in comparison to smaller primary tumours (first and second tertiles at baseline) at 3 mo (*p* = 0.9) or 6 mo (*p* > 0.9; Mann-Whitney *U* test).

**Fig. 3:**
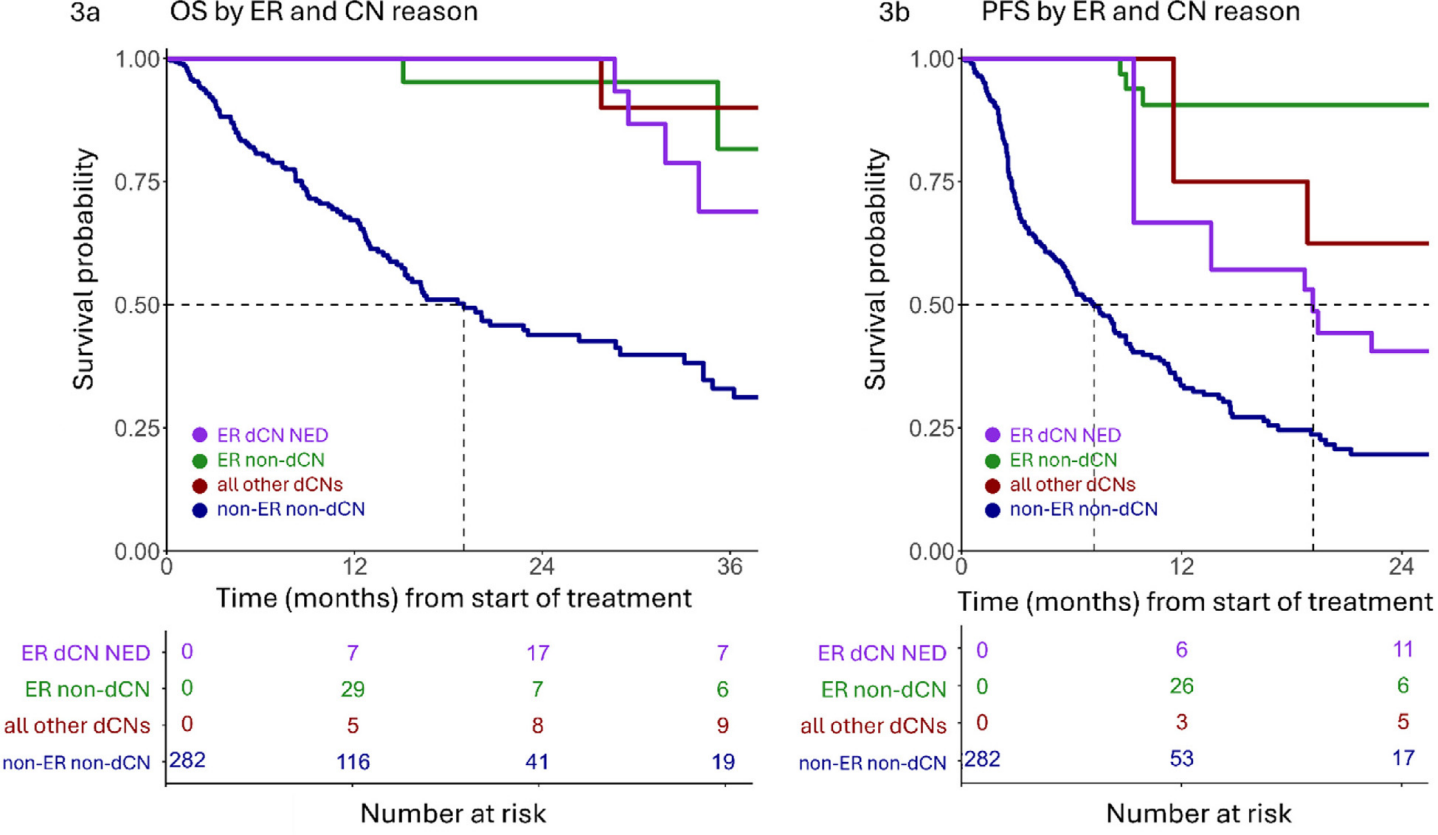
Extended Kaplan-Meier plots of (A) overall survival (OS) and (B) progression-free survival (PFS) over time, with exceptional response (ER; defined as a near-complete or complete response at metastatic sites) and deferred cytoreductive nephrectomy (dCN) modelled as time-dependent covariates, which can change in status over time. Each curve represents the estimated survival function for a hypothetical population whose values for these covariates remain fixed over time [25]. There were 128 OS events and 198 PFS events in the population evaluated; five ER patients (with one OS event and one PFS event in total) were not evaluated because the date for ER achievement was missing. ER dCN NED = dCN performed to achieve no evidence of disease in an ER case (27 of the cases evaluated) Ten of the ER cases evaluated underwent dCN for other reasons (six for primary tumour progression, four for primary tumour symptoms) and were counted with the No-ER dCN cases (n = 10) as “All other dCN cases”. For PFS only, dCN cases who underwent surgery after the date of first PD (n = 4) were considered no-dCN cases. Dotted lines at survival probability of 0.50 represent median survival. Plots were truncated when the number at risk in any group was <5 and was no longer increasing.

ER was achieved in 62 patients (22%), of whom 38 (13%) experienced CR and 24 (8.4%) experienced nCR. A total of 39 ER patients and ten non-ER patients underwent dCN (*n* = 49, 17%) at a median interval of 14.0 mo after starting N + I. The most common reason for dCN (Table 1) for ER patients was the depth of metastatic response (dCN with the aim of achieving NED, *n* = 27). OS and PFS, stratified by ER status and dCN status modelled as time-dependent covariates [25], are illustrated in Fig. 3. There was no significant difference in OS between the ER subgroup with dCN to achieve NED and the ER subgroup without dCN (HR 1.00, 95% CI 0.29–3.47; *p* > 0.9). dCN in four patients (8.2%) revealed a pathological CR; each patient was event-free for OS and PFS at last follow-up.

We stratified patients by inclusion criteria for the PROBE and NORDIC-SUN trials (Supplementary material) to project the expected OS in the non-dCN control arms. Trial randomisation at 12 wk and 3 mo, respectively, was used as time zero for landmark analysis. Patients who subsequently underwent dCN were censored at the time of surgery. Among patients alive at the randomisation landmark, 66% (*n* = 172) and 63% (*n* = 161) met inclusion criteria respectively, with post-landmark median OS of 36.8 mo (95% CI 30.3–not reached [NR]; Supplementary Fig. 1A) and 46.8 mo (95% CI 32.2–NR; Supplementary Fig. 1B) respectively.

## 4 Discussion

This s-mRCC cohort of patients treated with upfront N + I at five European centres provides real-world evidence of encouraging OS, especially in the intermediate IMDC risk group and in patients experiencing CR or nCR at metastatic sites.

A key strength of our study is the multi-institutional real-world approach using data from high-volume European renal cancer centres, and the inclusion of all N + I-treated patients with s-mRCC. Other series have involved highly selected dCN cohorts [10], [11], [12], [13], [14], [15], [16], [17], [18], [19], reported only landmark analyses [26], or pooled patients receiving N + I and patients receiving ICI/VEGFR combination regimens [27]. Our data allow contextualisation of dCN and ER rates and outcomes via comparison to the overall cohort. Limitations include the retrospective approach and the small size of certain subgroups for comparison, and our findings may not be generalisable to ICI/VEGFR regimens.

Our survival data, with median OS of 49.8 mo for the intermediate-risk group and 16.3 mo for the poor-risk group, are comparable to subgroup analysis [28] for the CheckMate 214 registrational trial of N + I [29], which showed median OS of 59.2 mo for the intermediate-risk group and 21.5 mo for the poor-risk group in a cohort of 53 patients treated with N + I for s-mRCC. The median OS of 29.0 mo for our overall cohort is in keeping with results for two real-world Japanese cohorts (25.3 mo, *n* = 33 [30]; 24.6 mo, *n* = 27 [31]). Our primary tumour PR rate was 30%, compared to 10% (three of 30) in an Italian cohort [24], 22% (five of 23) in a British cohort [32], and 35% (18/53) in CheckMate 214 [28]. Our dCN rate of 17% is identical to that in the CARMENA control arm (upfront sunitinib; 44% poor risk), suggesting a stable rate of clinical decisions for dCN from the VEGFR era to the ICI era.

Clinical experience suggests that N + I is less effective in controlling aggressive, high-burden disease in comparison to ICI/VEGFR regimens [33]. Primary refractory disease (best response PD) was observed in 33% of our patients (disproportionately IMDC poor risk), and we found significantly worse PFS and non-significantly worse OS for patients with four or more sites of metastasis at baseline. It is thought that N + I is highly active in sarcomatoid RCC [34]; we did not find an association between sarcomatoid histology and better OS or PFS, but without a comparator arm this may reflect the prognostic adversity of sarcomatoid histology over its predictive benefit for N + I. Other authors have found that larger tumours were less likely to respond by 6 mo, which suggests a role for upfront CN in these cases [24], but our data did not support this.

According to our data, dCN to achieve NED was not associated with a statistically significant survival advantage in the ER group; in fact, durable OS was observed even among dCN cases not resected to NED, but interpretation is limited by small subgroup sizes and selection bias. Two large prospective randomised trials comparing ICI combination regimens with and without dCN are currently recruiting: NORDIC-SUN [8] and PROBE [7]. NORDIC-SUN includes patients with s-mRCC treated with N + I or other ICI combination regimens, randomising only those who have three or fewer IMDC risk factors, adequate performance status, and no extrarenal progression at 3 mo, with a second evaluation at 6 mo for patients who are initially ineligible. The primary endpoint is a 50% improvement in OS in the dCN arm. The PROBE design is similar, with randomisation at 12 wk. These trials will clarify important questions; achieving NED status in patients is intuitively appealing, but its benefit here is as yet unproven.

The design of such trials has difficult practical and statistical challenges [9]. Importantly, long-term OS data following ICI treatment were not available when PROBE and NORDIC-SUN were designed; both statistical plans use OS data from the VEGFR era, with post-randomisation median OS estimates for the no-dCN control arms of 25.0 mo [7] and 24.0 mo [8]. In our cohort, the post-landmark median OS for patients who were alive at the same randomisation landmark was 30.2 mo, but crucially, inclusion criteria for these trials selected patients with good performance status, lack of early PD, and, for NORDIC-SUN, superior IMDC risk scores. After stratification of our cohort using these inclusion criteria, post-landmark median OS was 36.8 mo using the PROBE criteria, and 46.8 mo using to the NORDIC-SUN criteria. The absolute OS benefit of dCN implied by the design of the primary endpoints in these trials (improvements of 47% and 50%, respectively) is therefore greater than expected; using our data, this would be concordant with a median OS benefit of at least 17.3 mo and 23.4 mo, respectively. In addition, the event rate is expected to be lower with longer OS, which may mean that current sample sizes are inadequate.

This projection has several limitations. First, dCN cases were included to avoid immortal time bias, but were subsequently censored at the time of surgery; this avoids contamination but introduces informative censoring, with underestimation of OS. Second, our study population was less highly selected than in PROBE and NORDIC-SUN, as data for since some inclusion parameters—surgical fitness and other comorbidities, investigator opinion of pseudo-progression, and the NORDIC-SUN reassessment of eligibility at 6 mo—were not available in our data set. Together, these factors suggest that OS in the no-dCN control arms of PROBE and NORDIC-SUN may be even longer than we have calculated.

Finally, the interval between treatment initiation and surgery in PROBE and NORDIC-SUN of 3–6 mo (with a further 2 mo for surgical planning) is considerably shorter than the median interval in our real-world cohort (14.0 mo) and in the CARMENA control arm (11.1 mo). We note that the recently published protocol for the PrimerX trial [35] assumes median OS of 36 mo for patients without progression at 6 mo, and allows for randomisation to surgery between 6 and 18 mo.

Beyond existing trials, significant questions remain. If dCN would confer a benefit in some patients, can clinical, radiological, or biomarker parameters identify this population before surgery? What is the optimal interval from treatment initiation to surgery? For how long should maintenance nivolumab be continued, and could dCN reduce this treatment duration?

## 5 Conclusions

In conclusion, we found that N + I treatment of s-mRCC results in durable survival, especially in the group with intermediate IMDC risk. The role of dCN remains unclear; prospective randomised trials will need to be adequately powered to determine whether OS benefits exist. Further collection and analysis of real-world data are needed to investigate survival outcomes among ER and dCN cases, as well as the duration of maintenance nivolumab treatment in those achieving NED.

  ***Author contributions***: Leo Bickley had full access to all the data in the study and takes responsibility for the integrity of the data and the accuracy of the data analysis.

  *Study concept and design*: Bickley, Fransen van de Putte, Graafland, Haanen, Powles, Bex.

*Acquisition of data*: Bickley, Fransen van de Putte, van den Brink, Marandino, van der Mijn, Wilgenhof, van Thienen, Haanen, Boleti, Powles, Graafland, Turajlic, Bex.

*Analysis and interpretation of data*: Bickley, Bex.

*Drafting of the manuscript*: Bickley, Bex.

*Critical revision of the manuscript for important intellectual content*: Bickley, Fransen van de Putte, van den Brink, Marandino, Graafland, Wilgenhof, van Thienen, Haanen, Boleti, Powles, Zondervan, Graafland, Tippu, Turajlic, Bex.

*Statistical analysis*: Bickley, Bex.

*Obtaining funding*: None.

*Administrative, technical, or material support*: None.

*Supervision*: Bex.

*Other*: None.

  ***Financial disclosures:*** Leo Bickley certifies that all conflicts of interest, including specific financial interests and relationships and affiliations relevant to the subject matter or materials discussed in the manuscript (eg, employment/affiliation, grants or funding, consultancies, honoraria, stock ownership or options, expert testimony, royalties, or patents filed, received, or pending), are the following: Leo Bickley reports consultation fees from Telix. Sofie Wilgenhof reports institutional advisory board fees from Eisai, Bristol-Myers Squibb, Pfizer, Novartis, and Pierre Fabre. John B.A.G. Haanen reports advisory board roles for Achilles Therapies, BioNTech US, Gadeta, Immunocore, Neogene Therapeutics, Poke Acel, T-Knife, and Vazimm; advisory board attendance and/or lecture provision for Bristol-Myers Squibb, Instil Bio, Iovance Biotherapeutics, Merck Serono, Merck Sharp & Dohme, Novartis, Pfizer, and Sanofi; an advisory role for Bayer, Bristol-Myers Squibb, Eisai, Instil Bio, Iovance Biotherapeutics, Ipsen Bioscience, Merck Serono, Molecular Partners, Novartis, Pfizer, Roche/Genentech, Sanofi, and Seattle Genetics; institutional research funding from Amgen, Asher Bio, BioNTech US, Bristol-Myers Squibb, Merck Sharp & Dohme, and Novartis; an editor-in-chief role for ESMO Immuno-Oncology & Technology; and stock options in Neogene Therapeutics. Thomas Powles reports personal advisory board fees from Astellas, AstraZeneca, Bristol-Myers Squibb (BMS), Eisai, Exelixis, Incyte, Ipsen, Johnson & Johnson, Merck, Merck Serono, MSD, Novartis, Pfizer, Roche, and Seattle Genetics; personal travel grants from AstraZeneca, Ipsen, MSD, Pfizer and Roche; personal sponsorship for the Uromigos Podcast from Mashup; institutional honoraria from Gilead; and institutional research grants from Astellas, AstraZeneca, BMS, Eisai, Exelixis, Ipsen, Johnson & Johnson, Merck, Merck Serono, MSD, Novartis, Pfizer, Roche, and Seattle Genetics. Samra Turajlic reports speaking fees from Roche, AstraZeneca, Novartis, and Ipsen, and interests in patents for indel mutations as a therapeutic target and predictive biomarker (PCTGB2018/051892 and PCTGB2018/051893) and clear cell renal cell carcinoma biomarkers (P113326GB). Axel Bex reports grants from Pfizer; consultation fees from BMS, Roche, Ipsen, Eisai, Telix; and speaker bureau roles for Eisai and Ipsen. The remaining authors have nothing to disclose.

  ***Funding/Support and role of the sponsor*:** None.

  ***Acknowledgments:*** We wish to thank Hyung Kim and Ulka Vaishampayan for kindly providing insights into the design of the PROBE trial.
