# Corrigendum to “Long-term real-world survival outcomes with dual immune checkpoint blockade in synchronous metastatic renal cell carcinoma: implications for the design of prospective cytoreductive nephrectomy trials” [Eur. Urol. Open Sci. 83 (2026) 133–141]

**DOI:** 10.1016/j.euros.2026.05.009

**Published:** 2026-06-06

**Authors:** Leo Bickley, Elisabeth E. Fransen van de Putte, Luna van den Brink, Laura Marandino, Johannes C. van der Mijn, Sofie Wilgenhof, Johannes V. van Thienen, John B.A.G. Haanen, Ekaterini Boleti, Thomas Powles, Patricia J. Zondervan, Niels M. Graafland, Zayd Tippu, Samra Turajlic, Axel Bex

**Affiliations:** a Specialist Centre for Kidney Cancer, Department of Urology, Royal Free London NHS Foundation Trust, London, UK; b Cancer Dynamics Laboratory, Francis Crick Institute, London, UK; c Department of Urology, Netherlands Cancer Institute, Amsterdam, The Netherlands; d Department of Urology, Amsterdam UMC, University of Amsterdam, Amsterdam, The Netherlands; e Department of Imaging & Biomarkers, Cancer Centre Amsterdam, Amsterdam, The Netherlands; f Renal and Skin Units, Royal Marsden NHS Foundation Trust, London, UK; g Department of Medical Oncology, Netherlands Cancer Institute, Amsterdam, The Netherlands; h Specialist Centre for Kidney Cancer, Department of Medical Oncology, Royal Free London NHS Foundation Trust, London, UK; i Barts Cancer Institute, Queen Mary University, London, UK; j Renal Cancer Network, Amsterdam, The Netherlands; k Division of Surgery and Interventional Science, University College London, London, UK

The authors wish to correct the values given for duration of response in Results, paragraph 4. These were calculated incorrectly due to a coding error during analysis.

The values given for median duration of response were 8.0 mo (95% CI 5.3–9.9) among patients with response at metastatic sites and 6.4 mo (95% CI 2.9–12.0) among patients with response at the primary tumour. The correct values are 24.3 mo (95% CI 14.3 – NR mo) and 27.3 mo (95% CI 14.3 - NR mo) respectively.

The authors apologise for any inconvenience caused, and affirm that this corrigendum does not affect the conclusions of the article.

