# Supplementary material for: Long-term Real-world Survival Outcomes with Dual Immune Checkpoint Blockade in Synchronous Metastatic Renal Cell Carcinoma: Implications for the Design of Prospective Cytoreductive Nephrectomy Trials
Source: Eur Urol Open Sci. 2025 Dec 19;83:133–41. doi: 10.1016/j.euros.2025.12.004 (PMC12795699; doi:10.1016/j.euros.2025.12.004)
Supplement: Supplementary Data 1 [file mmc1.docx]

**Supplementary Material**

For survival analysis, stratification was performed by baseline or post-baseline variables. For post-baseline variables, immortal time bias was mitigated by use of landmark analysis, or (for dCN status, due to highly variable timing) time-dependent covariate modelling with visualisation by an extended Kaplan-Meier estimator^25^. Time origin was the date of first N+I dose unless otherwise stated. Patients were right-censored at administrative data cut and loss to follow-up. For PFS, death from any cause was considered an event.

Patients who died before first follow-up imaging assessment were excluded from best response analyses, and considered non-responders for overall response rate (ORR) and exceptional response (ER). Patients with missing data for date of best response (n = 63) were excluded from time to best response and duration of response; among these, exceptional responders with missing data for date of best response (n = 5) were also excluded from time-dependent modelling for OS and PFS according to ER and dCN status.

For projections relating to PROBE^7^ and NORDIC-SUN^8^ (Supplementary Fig. 1), OS was estimated by Kaplan-Meier landmark analysis, stratified by trial inclusion criteria: for PROBE, ECOG PS ≤ 1 with no active brain metastases and no extra-renal PD, CR or death before 12 weeks; for NORDIC-SUN, ≤3 IMDC risk factors at baseline, ECOG PS ≤ 1 and no extra-renal PD or death before 3 months. Date of trial randomisation (12 weeks and 3 months respectively after first N+I dose) was used as time origin for landmark analysis. To avoid immortal time bias, dCN patients whose surgery was performed after this landmark (in practice, all dCN patients studied) were considered includable in PROBE and NORDIC-SUN projections providing above stated inclusion criteria were met, but were then censored at time of surgery.

**
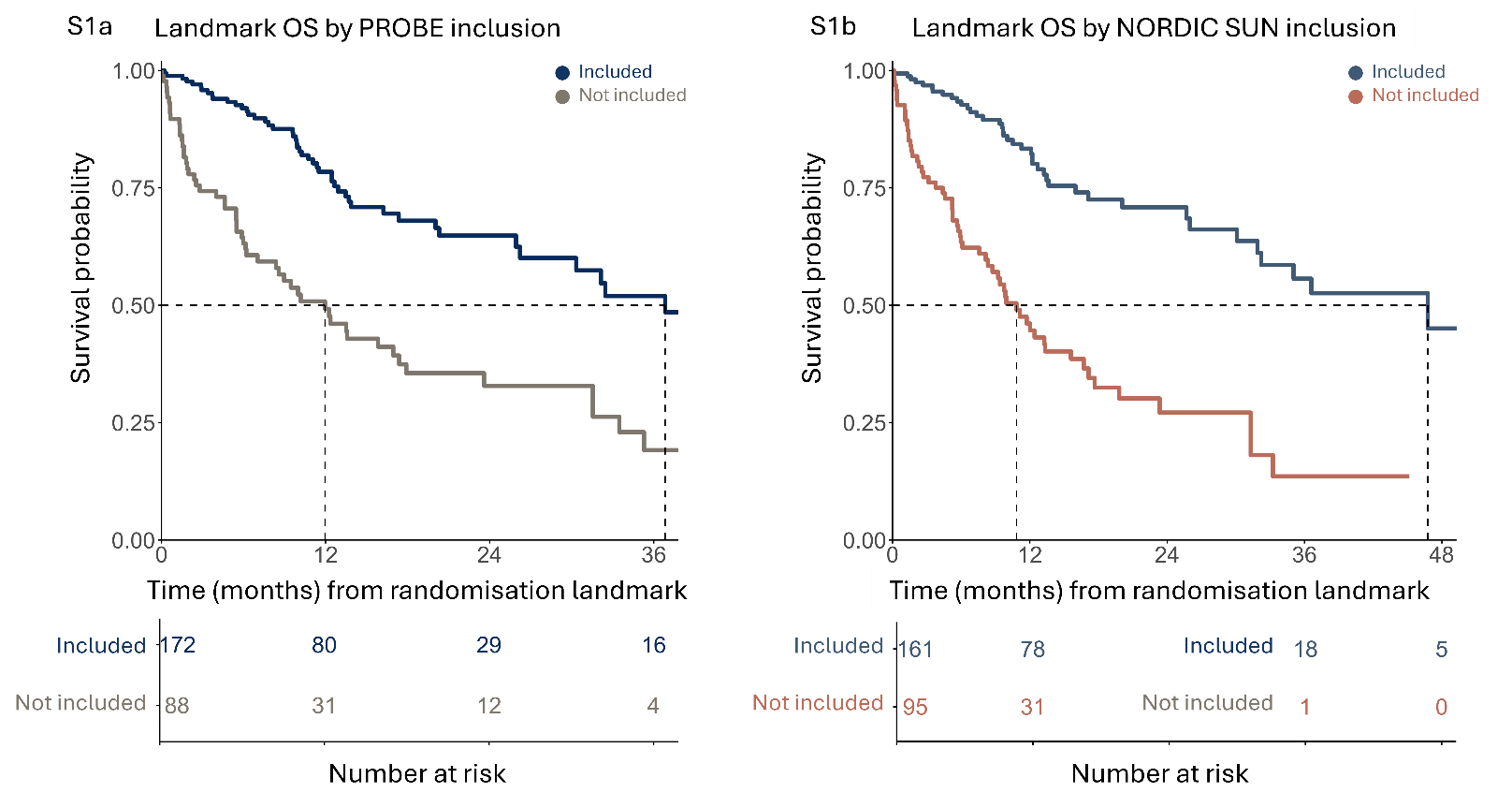
**

**Supplementary Fig. 1** – Kaplan-Meier plots over time (months), stratified by whether patients would have met inclusion criteria for (a) PROBE^7^ (ECOG PS ≤ 1 with no brain metastases and no extra-renal progression or CR before 12 weeks) or (b) NORDIC-SUN^8^ (≤3 IMDC risk factors, ECOG PS ≤ 1 and no extra-renal progression before 3 months). Trial randomisation at 12 weeks (a) or 3 months (b) was used as time zero for landmark analysis, to account for immortal time bias; 27 (a) and 31 (b) patients had OS events before this landmark and were not analysed. Patients that underwent dCN after this landmark (all dCN patients in our cohort) were includable in this analysis, but subsequently censored at time of surgery. Among analysed patients, there were 102 (a) and 99 (b) OS events. Dotted lines at survival probability of 0.50 represent median survival. Plots truncated when number at risk in the “included” group < 5.
